# Missing moments and being a burden: the lived experience of fear of cancer recurrence among older rural Australian women

**DOI:** 10.1007/s00520-026-10967-6

**Published:** 2026-07-10

**Authors:** Senara Kulatunga, Stacey Rich, Irene Blackberry, Christopher B. Steer

**Affiliations:** 1 School of Clinical Medicine, Rural Clinical Campus, University of New South Wales, Albury, Australia; 2 John Richards Centre for Rural Ageing Research, La Trobe University, 133 McKoy St Wodonga, Victoria, Australia; 3 Care Economy Research Institute, La Trobe University, 133 McKoy St Wodonga, Victoria, Australia; 4 Border Medical Oncology and Haematology, Albury Wodonga Regional Cancer Centre, Borella Rd Albury, New South Wales, Australia; 5 Border Medical Oncology Research Unit, Albury Wodonga Regional Cancer Centre, Borella Rd Albury, New South Wales, Australia

**Keywords:** Fear of cancer recurrence, Older women, Qualitative, Survivorship, Geriatric oncology, Rural

## Abstract

Fear of cancer recurrence is a common experience in cancer survivorship and can impact both physical and mental well-being. Some evidence exists on the manifestation of fear of cancer recurrence and its treatment. The purpose of this qualitative study was to fill a gap in the understanding of rural older women’s lived experience of fear of cancer recurrence. A sample of ten women aged ≥ 65 years living in rural Australia who were in remission from gynaecological cancers participated in semi-structured interviews about their lived experience of fear of cancer recurrence. Inductive thematic analysis generated four overarching themes that were subsequently mapped onto the Lee-Jones Model of fear of cancer recurrence, which provided a largely congruent interpretative framework for the affective, behavioural and cognitive aspects of older women’s experiences in this sample. However, themes relating to coping mechanisms and help-seeking behaviour extended beyond the model’s constructs, particularly around participants' strong self-reliance values and aversion to seeking support. Across interviews, a prominent concern was the fearof being a burden to family if cancer were to return, or concern for family members if they were to die. Further, they expressed an aversion to seeking professional help when worries about recurrence emerged, preferring to distract themselves or avoid thoughts of fear. The aversion to help-seeking highlighted in this sample suggested that traditional forms of support offered for fear of cancer recurrence may not be appropriate for older women. Further work is needed to establish targeted support for this cohort.

## Introduction

Cancer survivorship brings with it a burden of psychological work, including the fear that cancer may one day return. Fear of Cancer Recurrence (FCR) is defined as the “fear, worry, or concern about the possibility that cancer will come back or progress” p. 3266 [1]. FCR exists on a spectrum from normal to clinical levels [1]. Clinical FCR is characterised by high levels of preoccupation, high levels of worry, and hypervigilance to bodily symptoms [2]. Given these elevated levels of distress, FCR can significantly affect patients’ psychosocial well-being and physical health [3]. Improved screening and treatment strategies in the past two decades have led to increased survival rates among patients with cancer [4], resulting in more patients living in remission with the psychological burden that cancer may return.

**Fig. 1:**
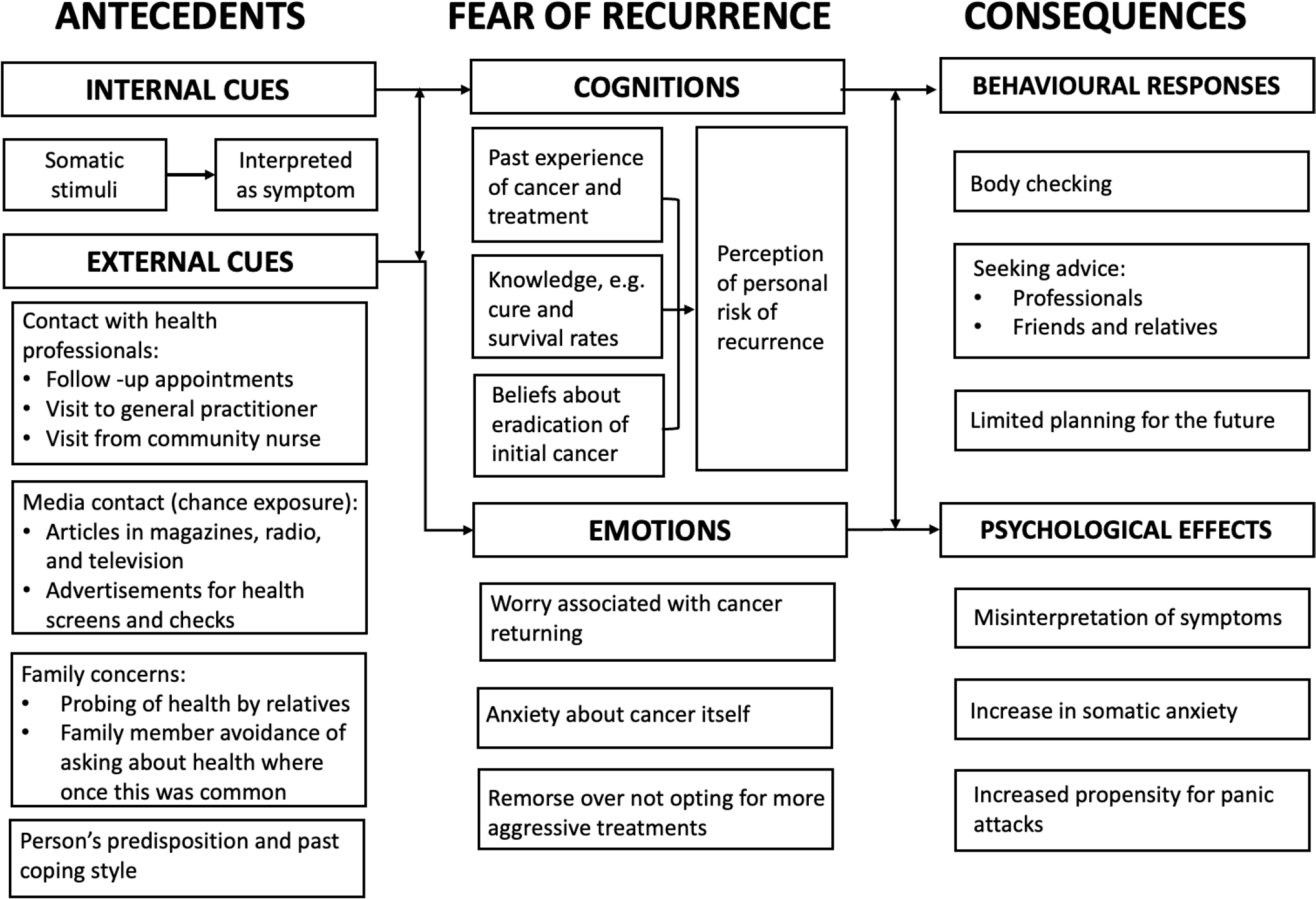
Lee-Jones' (1997) original summary of Fear of Recurrence (Agreement to use this image was provided on 17.12.2025, license 6,171,621,232,277)

The Lee-Jones model of Fear of Cancer Recurrence [5] suggests that internal and external cues trigger specific thoughts and emotions, leading to behavioural responses and psychological effects (Fig. 1). FCR severity can be influenced by pre-existing psychological issues, social support, sex, and age. Younger age and female sex have been identified as predictors of FCR [6].There is more evidence exploring FCR in younger people, such as young breast cancer [7–9] and childhood cancer survivors [7], than in older adults, especially women in rural areas.

For some cancers, the higher likelihood of recurrence contributes to a fear of recurrence grounded in a significant risk. Gynaecological cancers, especially ovarian cancer, are often diagnosed at advanced stages and are characterised by a high likelihood of recurrence following primary treatment [8]. Moreover, there is an inverse relationship between the age at diagnosis and 5-year survival rates [10]. Due to these significant prognostic risks, the uncertainty surrounding the disease trajectory can intensify fear of cancer recurrence in patients with gynaecological cancers [2, 8], making them a critical focus for study.

Psychological interventions appear to have a positive impact on FCR. In their meta-analysis of the effect of psychological interventions on FCR, Tauber et. al [11] noted a small but robust positive effect maintained at follow-up. However, a meta-regression within the same study found no significant moderating effect of age on intervention outcomes across samples with mean ages ranging from 44 to 70 years. This suggests that assumptions about lower FCR or intervention responsiveness in older adults may not be well supported. Absent from this evidence base are adults over the age of 70. Further, no studies have specifically examined the lived experience of FCR of older rural women with gynaecological cancers, a group that faces both distinct prognostic risks and barriers to accessing psychological support. Evidence suggests a prevalence of 16–19% of high FCR in older women [12]. Furthermore, and consistent with findings from the present study, the reluctance of older women to be perceived as a burden may lead to underreporting of FCR in self-report measures. This may lead to quantitative prevalence estimates not fully capturing the extent of distress experienced by this group. Qualitative approaches may offer a valuable complement to existing quantitative evidence.

This study examined the lived experience of FCR in older women in remission from gynaecological cancers, using inductive thematic analysis. The Lee-Jones model [5] was subsequently applied as an interpretative lens to situate the findings in the existing literature Where findings extended beyond the model's constructs, these are discussed in relation to the utility and limitations of conventional FCR frameworks and interventions for this cohort.

## Methods

### Design

This study employed a qualitative design using semi-structured interviews to explore the experiences of older women with gynaecological cancer in relation to fear of cancer recurrence (FCR). Data were analysed using reflexive thematic analysis following the six-step process described by Braun and Clarke [13]. This approach was selected as the most appropriate method for generating rich, participant-centred accounts of a complex psychosocial phenomenon, and is consistent with a constructivist epistemological position that acknowledges the role of the researcher in knowledge production.

### Participants and setting

Participants were women aged 65 years and older who had received treatment for epithelial ovarian cancer or uterine cancer at the Albury Wodonga Regional Cancer Centre (AWRCC) and were currently in remission. The decision to combine these two diagnostic groups reflected the similar FCR-related experiences associated with both cancer types and the small number of eligible patients available at this single regional centre. Inclusion criteria were: treatment at AWRCC, diagnosis of epithelial ovarian cancer or uterine cancer, current remission status, age 65 years or older, and ability to provide informed consent. The AWRCC serves a predominantly rural and regional catchment area, and all participants were therefore considered to meet the rurality criterion for the study.

### Recruitment and sample

**Fig. 2:**
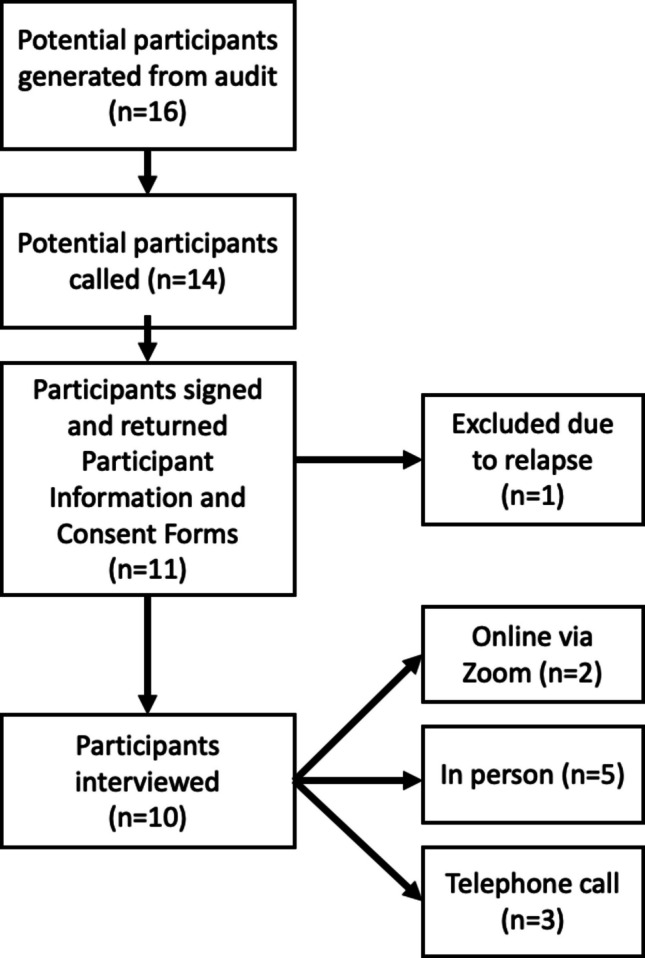
Participant recruitment flow

A convenience sample was drawn from patients under the care of Border Medical Oncology and Haematology at the AWRCC. Prospective participants were identified by the principal investigator (PI) from the eligible patient population. Participant recruitment is described visually in Fig. 2. To ensure representation of both diagnostic groups, participants were recruited with the aim of achieving an approximately equal distribution of ovarian and uterine cancer diagnoses, resulting in five participants with each diagnosis in the final sample.

Initial contact with all prospective participants was made by the student researcher or the second author who had no clinical relationship with the patient. The voluntary nature of participation was emphasised at each stage of the recruitment process: during initial contact, in the Participant Information and Consent Form (PICF), and again at the commencement of the interview. Participants were explicitly advised that their decision to participate or decline would have no bearing on their clinical care.

Consistent with the principles of reflexive thematic analysis, recruitment continued until data saturation was reached. Saturation occurred at ten interviews, at which point no new themes were emerging from the data. Although fourteen participants had been anticipated, the richness of the data and the specificity of the population were sufficient to achieve saturation with a smaller sample, consistent with recommendations for qualitative research with well-defined populations [13].

### Data collection

Once consent was received, each participant was asked to complete the fear of cancer recurrence inventory – short form [FCRI-SF: 13]. Participants completed the FCRI-SF nine-item Likert-type scale with response options ranging from 0 (“not at all” or “never”) to 4 (“a great deal” or “all the time”). A higher score represents a higher level of FCR, with possible scores ranging from 0 to 36. Clinical cut-off scores for the scale vary from > 13 [15] to ≥ 22 [16].

Participants also completed a demographics questionnaire that contained items pertaining to year of birth, employment/retirement status, category of previous/current work, highest level of education, perceived financial wealth (“In terms of financial wealth, I consider myself” (1) well below average to 5 (well above average)). Participants were also asked how many people lived with them, and their relationship to those people.

An interview schedule was used to guide discussion about affective (can you tell me a bit about worries you have about cancer recurring?), behavioural (when you worry about cancer recurrence, what do you do?) and cognitive (what most concerns you about cancer recurrence?) factors. The questions also tapped into specific examples of an episode of worry and help-seeking behaviours.

The first two interviews were conducted by the first author (SK, a student-researcher) under the supervision of the second author (SR), an experienced researcher. SK conducted the subsequent eight interviews independently. Interviews were conducted either face-to-face, via telephone or online using the Zoom digital platform (refer Fig. 2). Carers were encouraged to join the interviews, however, only one participant opted to have their carer present. Interviews were recorded using a digital voice recorder with the consent of the participant. Interview recordings were downloaded to a password-protected computer file and sent to a professional transcription service. Interviews ranged in duration from 24 to 75 min, with the average length being 46 min.

### Reflexivity

The PI held a dual role as both treating clinician for study participants and as principal investigator. To protect against potential bias arising from this, the PI did not conduct interviews, did not access interview transcripts, and played no role in data analysis or its supervision. Analytical supervision was provided independently by the research team at the La Trobe John Richards Centre for Rural Ageing Research (second author). This separation between the clinical and research functions of the study was maintained throughout the research process.

### Data analysis

Data analysis proceeded in two stages. In the first stage, the student researcher conducted an inductive thematic analysis of interview transcripts following the six-step framework of Braun and Clarke [13], generating codes and themes directly from participant accounts. This process produced four overarching themes: FCR influences and triggers; cancer recurrence consequences; FCR coping mechanisms; and help-seeking behaviour. Analytical rigour was supported through cross-coding of a subset of 4 interviews by SR with coding differences discussed and resolved through meetings. Supervision also included debriefing and reflexive discussion of emerging themes and occurred throughout the analysis period. NVivo (version 10.1.0) was used to support data management.

In the second stage, the four inductively generated themes were mapped onto the cognitive model of FCR described by Lee-Jones et al. [5]. This model was selected as the theoretical framework that best represented emergent findings and provided the most useful interpretive lens for situating results within the existing FCR literature. This mapping was largely congruent across the four themes. However, themes relating to coping mechanisms and help-seeking behaviour, particularly the autonomy-oriented sub-themes of independence, privacy, and help-seeking aversion, extended beyond the constructs in the Lee-Jones model [5]. These are interpreted accordingly in the Results and Discussion sections.

### Ethics

Ethical approval was granted by Albury Wodonga Health Human Research Ethics Committee (Approval no. 511/22/15). All participants provided written consent to participate and for their de-identified responses to be used in the dissemination of findings, including peer-reviewed publications. Participants were advised of the voluntary nature of participation and that they could choose to stop the interview at any time and withdraw their data. To protect participant privacy and confidentiality, all names were replaced by pseudonyms.

## Results

Ten women in remission from ovarian (*n* = 5) or uterine (*n* = 5) cancer were interviewed. The participants’ mean age at the time of diagnosis was 71 years, and at the time of interviewing, 74.4 years. Most participants were from small rural towns in Australia. Table 1 highlights participants’ demographic characteristics. Forty percent of the women lived alone, with the majority perceiving their wealth as at least average. FCRI-SF scores are presented in Table 1. Using the clinical cut-off of ≥ 13 established by Simard and Savard [15], six participants displayed clinically significant FCR and four did not meet this threshold. A more recent evaluation by Fardell et al. [16] suggested a higher cut-off of ≥ 22 may more accurately identify clinical FCR in English-speaking samples, applying which two participants in the current study would meet the clinical threshold. However, as Fardell et al. noted, younger age was associated with clinician-rated clinical FCR in their sample, and their study 1 participants were considerably younger (mean age 53 years, SD 10.1) than those in the current study. Given the ongoing debate regarding the appropriate clinical cut-off for the FCRI-SF and the limited validation data available for older adult populations, FCRI-SF scores are reported descriptively and interpreted with this limitation in mind.

**Table 1:** Demographic characteristics of participants and their fear of cancer recurrence index (short form) scores

Pseudonym	Diagnosis	Age at diagnosis	Age at interview	Education level	Perceived financial wealth	No. of home occupants	Relation to occupants	FCRI-SF score
Anne	Ovarian	72	76	< Year 12 or equivalent	Average	2	Partner/spouse	12
Bella	Uterine	69	71	Undergraduate diploma	Average	1	Lives alone	19
Cristy	Uterine	74	78	< Year 12 or equivalent	Well below average	1	Lives alone	11
Danni	Ovarian	81	86	< Year 12 or equivalent	Average	2	Partner/spouse	5
Eliza	Ovarian	66	68	< Year 12 or equivalent	Average	1	Lives alone	23
Fran	Uterine	66	69	< Year 12 or equivalent	Average	2	Partner/spouse	9
Greta	Ovarian	73	79	< Year 12 or equivalent	Average	1	Lives alone	26
Hanna	Uterine	65	67	Undergraduate diploma	Above average	2	Partner/spouse	19
Isa	Ovarian	70	71	Year 12 or equivalent	Average	2	Partner/spouse	17
Jane	Uterine	74	79	< Year 12 or equivalent	Average	7	Spouse, children and other relatives	16

**Fig. 3:**
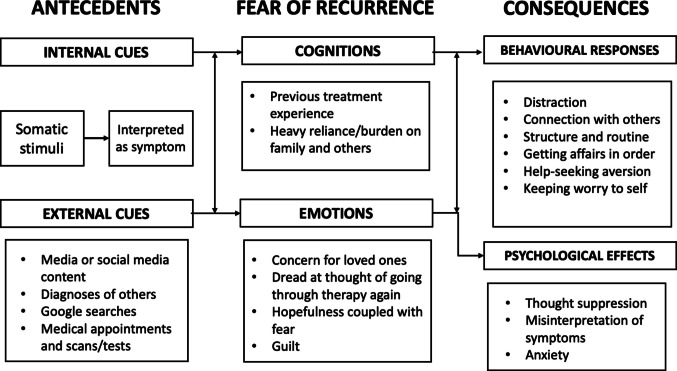
The Lee-Jones (1997) model of Fear of Cancer Recurrence, with examples highlighted in the current sample

Following inductive thematic analysis, the four overarching themes generated from participant interviews were mapped onto the Lee-Jones model [5] to aid interpretation, as illustrated in Fig. 3.

Participant experiences were congruent with those hypothesised by the Lee-Jones Model. One participant provided an example of that fit within one quote:

Again, that usually happens when I have a little – where’s that coming from, or what’s that? Or I have a rash on my knees, and I’m thinking, somebody said once if you had chemo, you’ll get skin cancer. I’m thinking, is that skin cancer on my knees? Then somebody said, that’s probably chilblains [laughs]. Those kind of things, they’ll trigger it. Then you think, hm. Yeah, or when I’m thinking of what’s going to happen to all this stuff? My son is in America. Do I start sending things, which for a while I started sending stuff over to him. – Bella, 71.

However, themes relating to coping strategies and help-seeking behaviour extended beyond the constructs described in the Lee-Jones model and are interpreted below.

### Antecedents

#### Internal cues

Many participants described how physical sensations and changes, such as pain or discomfort, immediately led to thoughts of cancer (Table 2). Even minor symptoms, like a twitch, rash, or general feeling of unwellness, were potent triggers that caused the women in this sample to worry about the potential return of cancer. These physical triggers became a source of constant vigilance and anxiety (cognition and emotion).

**Table 2:** Components of the Lee-Jones Model with participant’s illustrative quotes

Model Component	Illustrative quote
**Antecedents**	
**Internal** Somatic Sensations	“Any time you have a little twitch here, a little twitch there, and you think, is that cancer? Is it just bloated, or what is it?” Bella, 71 “If I get a little niggle I think, oh God, what’s that? You know, is that going to be cancer?” Greta, 79
**External**	
Media/social media	“Well, probably the other night when it just popped up on Facebook, symptoms of pancreatic cancer. I had been sick that day – like physically sick – I’d vomited, and I thought, no, don’t read it, but I did. Stupid. I thought, yeah, geez, that’s what I’ve got, I’ve got that, I’ve got that.” Kristy, 78
Scanxiety	“The most fear I had about the cancer returning was just before the first PET scan. That’s the scariest one when you have that coming up and the doctor suggests we have a PET scan.” Isa, 71
**Fear of recurrence**	
**Cognitions and emotions**	
Concern for family	“Well, just how they’d cope without the poor old dear.” Danni, 86 “I just want to see my grandkids grow up, but I mean there is always going to be a reason why you want to stay, isn’t there.” Greta, 79
Past experiences	“The fear of having to have another operation, like, that would knock my socks off, I think…not after the last one, it was pretty horrendous, wasn’t it?” Eliza, 68 “Worry about whether I’m going to have a return, because I believe chemo nearly killed me, not cancer. Chemo nearly killed me.” Greta, 79 Facilitator:“Are there any other worries, like going through the chemotherapy again or going through the treatment again that concerns you?” Interviewee:“Not really. The chemo, the people that are oncology are just marvellous, you know, like, you didn’t think oh god I’ve got chemo again because they were just so—weren’t they [daughters name]?” Eliza, 68
**Consequences**	
**Behavioural responses**	
Distraction	“I try to distract myself, do something else.” Anne, 76 “I do crafty stuff. I play the piano. I go visit friends. I just keep going.” Danni, 86
Not dwelling	“So, you know, don’t sit there and be sad.” Bella, 71 “But I just, sort of, think why waste my time thinking about it if I, you know, like, I don’t want to ruin what… I’m ft at the moment.” Fran, 69
Help seeking	“The only person I would really talk to is my girlfriend who is… a health professional” Isa, 71 “We are now doing a cancer group that went through this…That’s what we do for each other. We put our arms around each other, and you’ve always got someone to listen to you…” Anne, 76 “… and especially my daughter, and I am very close to my daughter – she’s been my rock.” Fran, 69 “I’ve got a good family. Great friends. If I needed anything, I think I’d turn to them first.” Isa, 71 “Like it was just a type of district nurse come community nurse, and she was ringing each month to find out if I needed anything and let me know that I was to ring her straightaway if I had any worries or needed to talk to anyone.” Fran, 69
Help seeking aversion	“I just feel – well, I don’t know the right word – but I hate asking for help. I hate asking for anything” Danni, 86 “I try and use all my own strategies on how not to be worried about things, yeah. So, yes, I like to do it myself rather than ask for help, probably.” Greta, 79

#### External cues

External information, such as news stories, books, or social media content related to cancer also served as a trigger (Table 2). Participants noted that consuming such information often led to heightened fears and concerns about their own health. Participants described how searching for health information online, including through platforms such as Google, often exacerbated rather than alleviated their fears, as described in Table 2.

Medical appointments, tests, and scans were significant sources of anxiety, as they brought the possibility of bad news to the forefront. The waiting period between tests and receiving results was particularly stressful, as it forced participants to confront the uncertainty of their health status. The act of seeing a doctor could stir up underlying fears, making the visit itself a trigger for anxiety.

### Fear of recurrence

Discussions with the older women in the sample revealed a complex interplay of emotions and cognitions. While some discussed fear of death or discomfort associated with treatment, overwhelmingly, the fear was rooted in concern for their loved ones. Despite the fear and anxiety associated with their illness and survivorship, many participants demonstrated resilience and a pragmatic approach to their circumstances. Some participants showed a level of acceptance regarding the possibility of dying, although this was often accompanied by concerns about leaving families behind.

I’m not frightened of dying, but I don’t want to leave my kids. It’s going to happen one day, but – yeah. Anne, 76

#### Concern for loved ones

A prominent theme across the interviews was participants’ deep concern for their loved ones, particularly in the context of their illness and potential recurrence. All participants discussed a reliance on family members for practical and emotional support during their illness. It is this heavy reliance on family for help during the initial illness that led to participant’s worries about the impact of their illness on their family members, often prioritising the well-being of others over their well-being. Many participants expressed a sense of guilt for the burden their illness placed on their families. For example, one participant worried about the pressure cancer put on her family and preferred to deal with recurrence worries herself to avoid causing stress to her husband. Participants also expressed concern about their family’s ability to cope in the future, especially in the event of their death or if cancer returned. This was reflected in worries about who will care for family members, particularly those who are vulnerable, such as children or spouses with health issues.

The prominence of concern for loved ones as a driver of FCR, rather than fear of death or treatment, may reflect the life stage of this cohort. For the older women in this study the fear of recurrence appears to be not primarily personal suffering but the disruption of those relationships and the burden placed on others. This finding suggests that FCR in older women may be qualitatively different in character from that reported in younger cohorts, where concerns about career, young children, and personal goals are prominent.

#### Past experiences

Past experiences with illness, particularly the challenges of treatment, amplified the fear of recurrence. Participants recall the difficulties they faced during their initial treatment and expressed dread at the thought of going through it again (Table 2). For some participants, however, positive experiences of cancer care attenuated these fears, with the quality of clinical relationships providing reassurance rather than heightening anxiety, as illustrated by one participant's account of the oncology team in Table 2.

### Consequences

#### Behavioural responses

Distraction was a common response to the experience of FCR in our sample. Participants frequently engaged in various activities to keep themselves occupied, as a deliberate strategy to divert attention from distressing thoughts or emotions (Table 2). These activities ranged from hobbies like playing music, crafting, or walking, to social interactions such as visiting friends or participating in group activities. These activities were viewed as a mechanism to focus energy and minimise the mental space available for ruminating on negative or distressing thoughts. Some took a pragmatic approach to busying themselves by focussing on practical matters, such as organising their affairs (e.g., paying for funerals in advance).

The theme of “not dwelling” emerges as a significant psychological response among this sample of older women. Across the interviews, participants consistently emphasise the importance of moving forward, managing their thoughts, and not allowing fear or anxiety to dominate their lives (Table 2). Participants also believed that worry and anxiety were normal in their situation and did not require attention.

When participants did seek help, support came in the form of family, friends and professionals, with family support being most often cited (Table 2). Second to family was the support of friends. The cancer journey also brought new connections and support into participants’ lives in the form of other survivors. Such connections became important supports to participants due to the shared understanding of experience.

Due to the rural locations where participants resided, district nurses played an important role in the survivorship experience, with one participant highlighting that she “knew [she] could ring them [at] any time”. While some felt comfortable speaking to their cancer care clinicians about their worries, others appeared to compartmentalise these professionals to their physical symptoms, not discussing their emotional concerns when prompted with questions such as “how are you?”.

Of the ten women in the sample, eight expressed an aversion to seeking help in general, and especially when it came to discussing fear of cancer recurrence (Table 2). Some expressed that they had “always been that way” when it came to seeking help, and that seeking help did not come naturally to them. Many spoke for their generation on the matter, utilising phrases such as “we don’t ask for help”. One participant spoke directly of not wanting to seek help from a mental health specialist, proposing that this was generational:

Yeah, see, I’m not one that would go to a psychologist or anything like that. I don’t know, I’m probably from a generation that, you know, you just get on with it. But apart from my doctor, I’d say no. Hanna, 67.

This pattern of self-reliance and help-seeking aversion, alongside participants’ expressed desire for independence, privacy, and autonomy, represents a cluster of findings that extends beyond the behavioural response constructs of the Lee-Jones model and has direct implications for how FCR support might be designed and delivered for this cohort.

## Discussion

This is the first contemporary study to investigate the lived experience of fear of cancer recurrence in older women in rural Australia. In line with the Lee-Jones model of FCR, the older women in this cohort reported both internal and external cues as triggers for their worry. For example, the group widely reported changes in bodily sensations triggering FCR. This is consistent with findings across age groups [17] and in different cancer types. Hearing of the cancer experiences of others also triggered FCR in this cohort. Psychosocial resources, such as family members and friends [2], were a helpful buffer to FCR for some of the participants in this cohort, as was their experience of their previous treatment. If their previous experience of treatment was positive, then some of the women felt they would be more able to cope with a recurrence in the future. The women also reported avoiding preoccupation with their fears and worries by keeping themselves busy. While avoidance coping strategies may work in the short term, their long-term use may be detrimental [18, 19].

Previous studies found that the content of FCR for women was often family-focused, with women in remission from cancer worrying about missing moments with children or grandchildren and wanting to protect their families from worry [20]. Particularly for middle-aged women diagnosed with ovarian cancer, fears and worries centre around how the family will cope without them [21], including financially. While some middle-aged women in Cesario et al.’s [21] study were worried about the financial strain on family and friends, the discussion of burdensomeness highlighted in our sample of older women was not evident in the content of fears in their sample.

A central theme to appear in the cognitions associated with FCR was that of burdening others if cancer were to return. Being a burden (or burdensomeness) appears to be a pervasive fear for older adults [22]. Further, feelings of burdensomeness are associated with depression and anxiety [23] and suicide ideation in older adults [24], suggesting that this aspect of FCR needs further attention in this age group. While participants in this study discussed fear of death, that fear was attached to missing moments with close others if they were to die rather than death itself. Further, the thought of undergoing treatment again appeared to be an important aspect of the affective component of FCR experience for this cohort, although positive experiences with previous treatment attenuated this.

While the experience of FCR in this cohort appears to fit the Lee-Jones model of FCR, the suitability of current interventions for this cohort is unclear. More contemporary theoretical frameworks, including the cognitive processing formulation proposed by Fardell et al. [25], extend the Lee-Jones model by highlighting metacognitions, worry and rumination as central maintaining mechanisms of FCR, and by highlighting vulnerability factors such as caring roles and previous losses. These constructs offer additional explanatory power for the patterns observed in the present study, where concern for loved ones and fear of becoming a burden were among the most prominent drivers of FCR. There is a growing body of evidence for the efficacy of FCR interventions [11]. However, those interventions may not be suitable for older rural women, such as those who participated in this study. Using the original clinical cut-off of ≥ 13 [15], six participants displayed clinically significant FCR, though as noted, the more recently proposed cut-off of ≥ 22 [16] would identify two participants at clinical threshold. Despite this, the older women interviewed were fiercely independent and appeared to have an aversion to seeking help when they worried about cancer recurring. These stoic and resilience characteristics are common among rural residents as formal or professional help is often difficult to access. For those happy to seek help, the first port of call for most of the women in this cohort was family. The preferred type and source of support varied across participants, consistent with evidence of variability in cancer survivors support needs [6]. Others found solace in support groups. This is in line with other research that suggests older adults are reluctant to seek explicit or professional help for psychological and mental health issues [26], with those in the 65 to 85-year age bracket accessing psychologist services at almost half the rate of their 16–34-year-old counterparts [27]. Reported barriers to older adults seeking help include thinking others need help more than themselves, believing anxiety and depression are normal states in older age, embarrassment associated with talking about their problems, and concern about burdening others with their problems [26]. This would suggest that referrals to mental health professionals and FCR programs may not be taken up by this cohort. The need to travel to larger towns due to workforce shortage may exacerbate this issue. However, given that older women do appear to experience FCR with a unique focus on burdensomeness, and older adults make up a significant portion of all cancer diagnoses, developing appropriate and flexible support mechanisms for this cohort will be critical.

### Strengths and limitations

The qualitative approach allowed for rich, detailed descriptions of participants’ experiences and emotions. Their freely expressed accounts of their experience capture nuanced perspectives, particularly on sensitive topics like dealing with cancer, that would be difficult to capture quantitatively. Further, interviews offered voice to the lived experience of the often-overlooked cohort of older rural women.

While qualitative studies typically have smaller sample sizes to allow for in-depth exploration, this limits the generalisability of findings. Given that our sample was situated in one rural Australian site and focused on a specific type of cancer, the participants’ experiences may not represent the broader population of cancer patients. While there was diversity in the sample concerning living arrangements, most had average to above-average income and similar educational attainment levels. There is always the risk of researcher bias in qualitative studies, especially in interpreting themes. Analytical rigour was supported through cross-coding of a subset of interviews by the second author. There was strong concordance between coders, with difference resolved through supervision.

The dual role of the principal investigator as both treating clinician and researcher represents a potential source of power imbalance in recruitment. As described in the Methods section, safeguards were implemented to mitigate this.

## Conclusion

Our qualitative analysis, in which inductively generated themes were subsequently mapped onto the Lee-Jones model of FCR, suggests the model broadly reflects the lived experience of older women with a history of gynaecological cancer. It also reveals dimensions of experience within the current sample, particularly burdensomeness, autonomy, and help-seeking aversion, that extend beyond the model’s constructs. While psychosocial resources, including family support and positive past treatment experiences, provide some relief, the fear of being a burden to others emerges as a prominent concern in this cohort. The aversion to seeking professional mental health support, common among older adults, underscores the need for tailored interventions that address their unique fears and preferences. Given the significant impact of FCR on quality of life, especially with concerns about burdensomeness and independence, it is crucial to develop appropriate and accessible support mechanisms for older women facing cancer recurrence fears.

## Data Availability

The data surveys that support the findings of this study are available from La Trobe University, but restrictions apply to the availability of these data, which were used under licence for the current study and so are not publicly available. The data are, however, available upon request and with the permission of La Trobe University.
